# Sleep quality moderated the mediating effect of BMI and waist circumference on the relationship between screen time and mental health in Chinese adolescents

**DOI:** 10.3389/fped.2025.1602512

**Published:** 2025-09-18

**Authors:** Yi Wang, Huipan Wu, Jian Wu, Yuanyuan Ma, Jinxian Wang

**Affiliations:** ^1^ School of Sport and Physical Education, North University of China, Taiyuan, Shanxi, China; ^2^ Research Center for Health Promotion of Children and Adolescents, Taiyuan Institute of Technology, Taiyuan, Shanxi, China; ^3^ School of Sports Science, Jishou University, Jishou, Hunan, China

**Keywords:** screen time, mental health, body mass index, waist circumference, sleep quality, mediating effect

## Abstract

**Objective:**

This study aimed to investigate whether body mass index and waist circumference can moderate the relationship between screen time and adolescent mental health, and whether sleep quality can moderate these effects.

**Method:**

Using a stratified cluster random sampling method, 5,713 adolescents aged 13–18 years were selected from six administrative regions in China for a questionnaire survey. Data analysis used statistical methods such as Pearson correlation analysis. The mediation model was tested using the PROCESS (version 3.3) SPSS macro model 4 developed by Hayes, and model 7 was used to test the moderation model.

**Results:**

Screen time was negatively correlated with adolescent mental health (*r* = −0.10, *p* < 0.001) and positively correlated with BMI (*r* = 0.03, *p* < 0.05). Screen time has a significant negative effect on adolescents’ mental health [*β* = −0.20, SE = 0.07, *p* < 0.01, 95% CI = (−0.34, −0.06)], while BMI mediates the relationship between screen time and mental health [*β* = −0.89, SE = 0.12, *p* < 0.001, 95% CI = (−1.11, −0.66)]. Additionally, sleep quality moderates the relationship between screen time and mental health in adolescents [*β* = 0.04, SE = 0.01, *p* < 0.001, 95% CI = (0.02, 0.07)]. There was no correlation between screen time and waist circumference (*r* = −0.005, *p* > 0.05), but there was a negative correlation between waist circumference and mental health (*r* = −0.04, *p* < 0.01). Waist circumference did not mediate the relationship between screen time and mental health (*p* > 0.05), nor did sleep quality moderate the relationship between screen time and waist circumference (*p* > 0.05).

**Conclusion:**

BMI plays a partial mediating role between screen time and mental health among Chinese adolescents. Additionally, sleep quality weakens the association between screen time and mental health. Therefore, it is recommended to alleviate mental health issues caused by excessive screen time among adolescents by promoting sleep quality.

## 1 Introduction

According to the latest report from the World Health Organization, one in seven adolescents aged 10–19 worldwide suffers from mental disorders, accounting for 13% to 15% of the global disease burden in this age group (1). This situation not only poses a threat to the future development of young people, but also presents a serious challenge to the overall health and sustainable development of society. Public awareness of adolescent mental health issues as an integral part of health and social development is growing, and global action on mental health is seen as a prerequisite for achieving the Sustainable Development Goals (2, 3). According to related reports, the overall prevalence rate of psychological disorders among Chinese adolescents is 17.5% (4). Compared with other age groups, adolescents have a higher incidence of mental health problems (5). The Chinese government attaches great importance to the mental health issues of adolescents and has adopted a series of policy measures to strengthen mental health services and mental health work (6, 7). Therefore, exploring risk factors and potential mechanisms related to mental health issues is of great theoretical and practical significance for the prevention of mental health issues among adolescents and the development of intervention measures.

Screen time is a type of sedentary behavior that includes watching television, using computers, using mobile phones, and other devices with electronic screens. The average daily screen time for adolescents worldwide generally exceeds the WHO recommendation of two hours (8). According to reports, 41.5% of Chinese adolescents spend more than two hours per day on screens during the weekend (9). Research shows that smartphones are the most widely used devices among adolescents, with widespread applications in social networking, learning, and entertainment, which are associated with mental health issues (10). A systematic literature review and meta-analysis found that smartphone addiction is significantly associated with a variety of mental health issues, including loneliness, sleep disorders, psychological distress, stress, and cyberhypochondria (11). Research indicates that different developmental trajectories of screen use among adolescents are differentially associated with mental health and behavioral outcomes in early adulthood (12). Another study found that excessive screen time among adolescents is associated with deteriorating social relationships and psychological stress (13). In addition, excessive screen viewing among adolescents may also be associated with internalization problems, mental health levels, and perceived quality of life (14). Based on the above findings, this study proposes Hypothesis 1: Screen time is significantly negatively correlated with adolescent mental health.

Body Mass Index (BMI) is an important standard for measuring a person's body fat percentage and overall health. Relevant data indicates that the prevalence of overweight and obesity among children and adolescents aged 6–17 in China has been increasing annually. It is projected that by 2023, the proportion of overweight and obese children and adolescents will reach 31.8% (15). According to Bruce McEwen's Allostatic Load Model (16), when individuals face long-term or repeated stressors, the physiological system produces an “overmobilization” response to maintain internal environmental stability. This long-term physiological activation can lead to sustained stress on the neuroendocrine-immune-metabolic system, manifesting as elevated inflammation levels, cortisol metabolic disorders, increased fat accumulation, and a series of other chronic health risk indicators, ultimately affecting mental health. Among adolescents, excessive screen time acts as a psychological stressor (17). Long-term, high-frequency screen exposure is accompanied by reduced physical activity and a decrease in resting metabolic rate, leading to obesity and an increase in BMI (18). A meta-analysis showed that adolescent obesity is positively correlated with increased use of electronic devices (19). In addition, adolescents who spend more than two or three hours a day watching television are at increased risk of obesity (20, 21). At the same time, excessive screen time among adolescents is associated with abdominal obesity (22). A study of Brazilian adolescents suggests that watching more than three hours of television per day may increase the risk of abdominal obesity (23). Overweight or obese adolescents are not only associated with excessive screen time, but also with mental health. Adolescents with high BMIs are at higher risk of developing depressive symptoms (24). Research shows that adolescents with higher BMIs are more likely to be subjected to verbal abuse, which leads to lower self-esteem and an increased risk of psychological problems. A study covering more than 1 million adolescents aged 11–15 worldwide found that there is a U-shaped relationship between adolescent BMI and mental health, with overweight and obesity significantly associated with higher levels of psychological distress (25). Based on the above research, most studies only use BMI as an indicator of obesity and explore the relationship between the two variables without investigating the underlying regulatory mechanisms. This study took into account that BMI cannot effectively assess body fat distribution, especially abdominal fat. Waist circumference, as a more direct indicator of abdominal fat accumulation, has been confirmed by research to improve the accuracy of assessing obesity and health risks when used in combination with BMI (26, 27). Therefore, this study combines BMI and waist circumference and proposes Hypothesis 2: BMI and waist circumference mediate the relationship between screen time and adolescent mental health.

Although an increasing number of studies have focused on the impact of screen time on adolescent mental health, there is still a lack of clear understanding of the underlying mechanisms. Problematic screen use may affect mental health through physical health indicators, but the mediating and moderating mechanisms remain unclear. In particular, the moderating role of sleep quality in the relationship between screen time, BMI/WC, and mental health has not been systematically studied (28, 29). Sleep quality refers to an individual's perceived satisfaction with their sleep (30). Sleep quality is not merely a matter of the length of sleep, but more importantly, the depth, continuity, and how one feels upon waking. According to self-regulation theory, good sleep quality can enhance an individual's self-regulation abilities (31), including cognitive regulation and behavioral regulation, thereby reducing the risk of obesity. Studies show that adolescents with poor sleep quality have significantly higher BMIs than those with normal sleep quality (32). A nationwide study in South Korea found that adolescents who sleep less than six hours a night are at a much higher risk of obesity than those who get adequate sleep (33). Good sleep quality promotes metabolism in adolescents and reduces the risk of obesity (34). Good sleep quality can regulate appetite hormones, reduce hunger, and increase satiety (35). In addition, sleep quality is directly related to fat oxidation efficiency at rest (36). Similarly, good sleep quality can enhance self-regulation, thereby reducing dependence on screens (37). Based on the above, this study proposes Hypothesis 3: Sleep quality moderates the relationship between adolescents' BMI and waist circumference and screen time.

**Figure 1:**
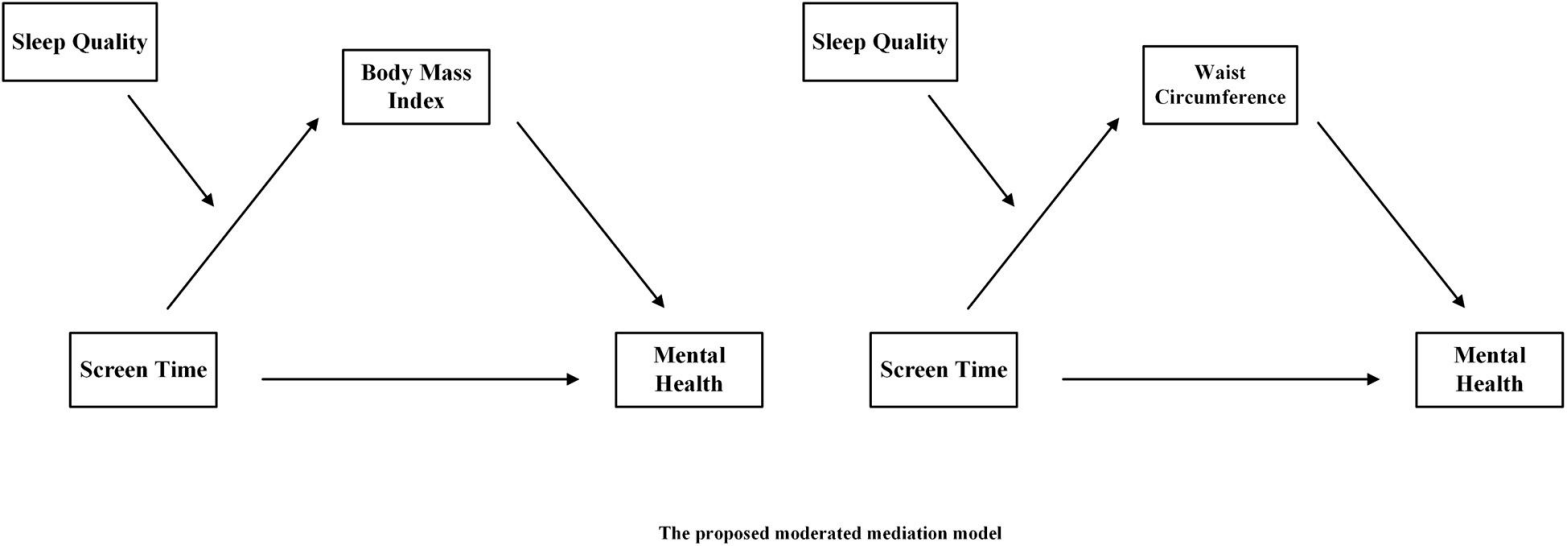
The proposed moderated mediation model.

In summary, this study employs a large-scale cross-sectional survey targeting Chinese adolescents. By introducing BMI and waist circumference as mediating variables and sleep quality as a moderating variable, and based on Bronfenbrenner's ecological systems theory, it provides a new theoretical perspective for exploring the complex relationship between adolescents' screen time and mental health. By revealing the potential mechanisms underlying the relationship between screen time and adolescent mental health, this study aims to enrich the theoretical foundation of this research field and provide scientific evidence for future interventions and related policy-making. Therefore, this study constructed two hypothesized path models (see Figure 1).

## 2 Research subjects and methods

This study is a cross-sectional design, which has limitations in causal inference compared to longitudinal studies. However, cross-sectional mediation analysis can still provide valuable preliminary evidence on the potential mechanisms through which screen time affects mental health via BMI and waist circumference, and test the moderating effect of sleep quality.

### 2.1 Research subjects

**Figure 2:**
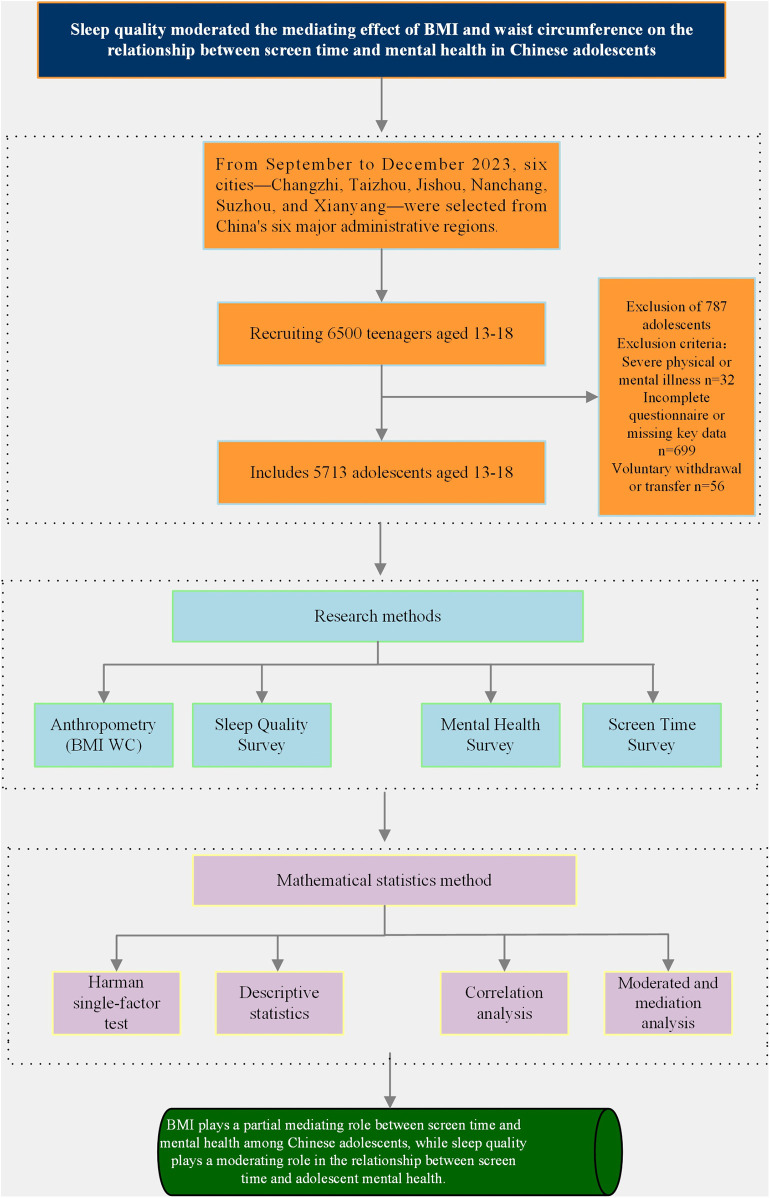
Research process.

This study was conducted from September to December 2023. Six cities—Changzhi, Taizhou, Jishou, Nanchang, Suzhou, and Xianyang—were selected based on China's six major administrative divisions. A stratified cluster random sampling method was used to conduct field tests and surveys in these six cities. To ensure a balanced gender and age distribution of the sample, a total of 18 secondary schools were selected across various cities. Using a random lottery method, 216 classes were selected from each grade, and all students were included in their respective classes. Prior to the formal investigation, each school will organize an offline briefing session, where testers will explain in detail to participants the purpose, process, potential risks, and data confidentiality mechanisms of the study. Both students and their guardians will be present and will voluntarily sign a written informed consent form. All questionnaires were in paper form, distributed on site by test administrators who explained how to fill them out, supervised the completion process, and collected them centrally. The inclusion criteria for this study were: students aged 13–18 who were currently enrolled in school, and both the participant and their guardian consented to participate in the study. Exclusion criteria included: participants with severe physical or mental health conditions that could potentially influence study outcomes, as well as those who completed the questionnaire incompletely or had missing key data. Comparisons of demographic characteristics between included and excluded participants showed no significant differences, supporting the assumption of random complete non-response. Statistical power analysis was conducted using R Core Team tools, determining the target sample size to be 954. A total of 6,500 questionnaires were distributed across six cities, with 5,713 valid responses, yielding a response rate of 87.89%. Among these, 2,908 were male and 2,805 were female, with an average age of 15.11 ± 1.70 years. This study was approved by the Human Experiment Ethics Committee of East China Normal University (HR761-2022). All participants and their guardians were informed of the study's purpose and signed informed consent forms. All procedures adhered to current ethical guidelines for research on adolescent mental health and screen time, and all data were anonymized and de-identified (see Figure 2).

### 2.2 Research methods

#### 2.2.1 Anthropometry

Measurements of height, weight, and waist circumference for adolescents are conducted by trained professionals using instruments of the same model, following standardized procedures. Height measurements are taken using a mechanical height gauge, with readings accurate to 0.1 cm; weight measurements are taken using a lever-type scale, with readings accurate to 0.1 kg. Normal weight, overweight, and obesity are evaluated according to the 2018 Chinese health standard “Screening for Overweight and Obesity Among School-Age Children and Adolescents” (38).

Waist circumference is measured using a tape measure, with the lower edge of the tape measure positioned 1 cm above the umbilicus. The measurement is taken by passing the tape measure horizontally around the waist, following the midpoint between the lower edges of the 12th ribs on both sides and the upper edge of the iliac crest, with readings accurate to 0.1 cm. P75 is used as the upper limit for normal waist circumference in children and adolescents, and P90 as the cutoff point for high waist circumference in children and adolescents (39). Waist circumference, height, and weight were each measured twice, and the average values were taken.

#### 2.2.2 Sleep quality survey

The Pittsburgh Sleep Quality Index (PSQI) (40) was used to assess sleep quality in adolescents. The PSQI consists of 19 self-rated items and 5 other-rated items, with the 19th self-rated item and the 5 other-rated items not included in the scoring. The 18 items are grouped into 7 components, each scored on a 0–3 scale. The cumulative score of all components constitutes the PSQI total score, ranging from 0 to 21. A higher score indicates poorer sleep quality. A total score ≤5 indicates good sleep quality, while >5 indicates poor sleep quality. This questionnaire has undergone rigorous psychometric validation, with Cronbach's *α* coefficients of 0.842 for each dimension and a test-retest reliability of 0.809, demonstrating good reliability and validity.

#### 2.2.3 Mental health survey

The mental health survey was conducted using the Brief Instrument on Psychological Health of Youths (BIOPHY) developed by Tao Shuman et al. (41). The questionnaire consists of 15 items across three dimensions: emotional problems, behavioral problems, and social adaptation difficulties. The item options range from “1 = lasting more than three months” to “6 = none or lasting less than one week.” Higher scores indicate shorter durations of mental health issues, with symptoms lasting one month or longer defined as indicative of suboptimal mental health. The P10 scale was used as the criterion for defining adolescent mental health issues. The results of factor analysis showed that the KMO statistic was 0.953, the Bartlett's sphericity test *P*-value was less than 0.01, and the cumulative variance explained was 57.39%. In terms of reliability analysis of the questionnaire, the Cronbach's *α* coefficient was 0.928, and the split-half reliability coefficient was 0.909, both meeting the reliability requirements for psychological research, indicating that the questionnaire has high consistency and reliability.

#### 2.2.4 Screen time survey

The “Physical Activity Level Evaluation for Children and Adolescents Aged 7–18 Years” was used to conduct a screen time survey among adolescents. The questionnaire primarily investigated the total screen time (watching TV/movies, using computers, mobile phones, and tablets) per week, excluding time spent on offline classroom instruction and online video instruction arranged by educational institutions, over the past week. The questionnaire has a test-retest reliability coefficient of 0.606, a correlation coefficient of 0.689, and *P*-values all <0.01, indicating good reliability and validity. This questionnaire has been widely applied in research and public health monitoring of Chinese adolescents and demonstrates excellent practicality.

### 2.3 Covariates

The study controlled for gender and age, taking into account the impact of demographic variables such as gender and age on the analysis results.

### 2.4 Mathematical statistics method

This study used Statistical Product and Service Solutions 26.0 statistical software to process and analyze the data. First, the software was used to check whether the research data contained common method bias. A critical value of less than 40% indicates that the study did not contain common method bias. Second, descriptive statistics and correlation analysis were performed on the relevant variables, using the PROCESS SPSS macro model compiled by Hayes (42) to examine the mediating effect of BMI and waist circumference on the relationship between screen time and adolescent mental health, and Model 7 to examine the moderating effect of sleep quality on the relationship between screen time and adolescent BMI and waist circumference. Controlling for gender and age, screen time as the independent variable, mental health as the dependent variable, BMI and waist circumference as mediating variables, and sleep quality as the moderating variable. To test the research hypothesis, the Bootstrap resampling method was used, with the number of resampling set to 5,000. If the 95% confidence interval (CI) did not include zero, the moderating mediating effect was determined to be statistically significant.

## 3 Results

### 3.1 Harman single-factor test

Research data collected through questionnaires are susceptible to common method bias. Therefore, Harman's single-factor test was used to examine common method bias. The analysis results showed that eight factors met the criterion of eigenvalue greater than 1. Without principal component factor rotation, the first factor accounted for 26.39% of the variance, which was below the critical value of 40% (43). Therefore, the analysis results indicate that there is no common method bias in this study.

### 3.2 Descriptive statistics

Table 1 shows the analysis of 5,713 adolescents, including 2,908 males (50.90%) and 2,805 females (49.09%). Significant differences were observed between adolescents of different genders in terms of screen time, BMI, waist circumference, mental health scores, and sleep quality scores (*p* < 0.001). Significant differences were also observed among adolescents aged 13–18 years in terms of screen time, BMI, waist circumference, mental health scores, and sleep quality scores (*p* < 0.05).

**Table 1:** Description of demographic characteristics.

Characteristic		Number of respondents (%)	Screen time		Body mass index		Waist circumference		Mental health		Sleep quality	
			M ± SD	T/F value	M ± SD	T/F value	M ± SD	T/F value	M ± SD	T/F value	M ± SD	T/F value
Gender	Male	2,908 (50.90%)	1.31 ± 1.78	4.49***	22.02 ± 4.61	6.10***	71.93 ± 26.89	10.45***	75.52 ± 14.24	6.51***	4.20 ± 2.62	−10.61***
	Female	2,805 (49.09%)	1.11 ± 1.59		21.29 ± 4.48		65.75 ± 16.30		76.94 ± 15.65		4.94 ± 2.63	
Age	13	1,265 (22.14%)	1.06 ± 1.56	10.43***	21.33 ± 1.89	9.83***	68.98 ± 26.25	2.86*	78.36 ± 15.33	2.78*	3.90 ± 2.49	40.76***
	14	1,362 (23.84%)	1.33 ± 1.90		21.21 ± 4.67		68.37 ± 16.94		77.22 ± 16.20		4.25 ± 2.81	
	15	752 (13.16%)	1.47 ± 1.93		21.81 ± 4.39		71.67 ± 27.00		77.66 ± 15.38		4.58 ± 2.81	
	16	801 (14.02%)	1.18 ± 1.61		21.85 ± 3.97		68.45 ± 19.98		78.73 ± 14.18		4.98 ± 2.48	
	17	908 (15.90%)	1.22 ± 1.59		21.89 ± 4.33		68.36 ± 21.49		79.05 ± 13.75		5.10 ± 2.37	
	18	625 (10.94%)	0.93 ± 1.31		22.56 ± 4.72		67.87 ± 23.55		79.56 ± 13.79		5.24 ± 2.59	

*p < 0.05.

**p < 0.01.

***p < 0.001.

### 3.3 Correlation analysis

Table 2 Pearson correlation coefficients between relevant variables. Screen time was significantly negatively correlated with adolescent mental health (*r* = −0.10, *p* < 0.01) and significantly positively correlated with BMI (*r* = 0.03, *p* < 0.05). The PSQI total score was significantly positively correlated with BMI (*r* = 0.18, *p* < 0.001). Adolescent BMI was significantly negatively correlated with mental health (*r* = −0.14, *p* < 0.001). Adolescent waist circumference was significantly negatively correlated with mental health (*r* = 0.04, *p* < 0.01), and waist circumference was not correlated with PSQI total score or screen time (*r* = 0.01, *r* = −0.005, *p* values both > 0.05).

**Table 2:** Pearson correlation coefficients among the relevant variables.

Variable	1	2	3	4	5	6	7
1 Gender	–						
2 Age	0.003	–					
3 Screentime	−0.06***	−0.02	–				
4 BMI	−0.08***	−0.09***	0.03*	–			
5 Wc	−0.13***	−0.01	−0.005	0.25***	–		
6 Menal health	−0.09***	0.03*	−0.10***	−0.14***	−0.04**	–	
7 PSQI	0.14***	0.18***	0.10***	0.18***	0.01	−0.50***	–

PSQIP, Piittsburgh Sleep Quality Index; WC, waist circumference; BMI, body mass index.

*p < 0.05.

**p < 0.01.

***p < 0.001.

### 3.3 A mediating model test

#### 3.3.1 Testing of the BMI intermediary model

**Figure 3:**
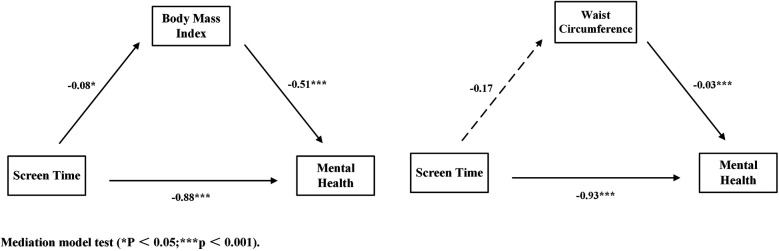
Mediation model test.

After controlling for covariates, screen time had a significant direct effect on adolescents' mental health [*β* = −0.88, SE = 0.12, *p* < 0.001, 95% CI = (−1.11, −0.66)], with a large effect size. When BMI was included as a mediating variable, the effect of screen time on adolescents' mental health remained significant [*β* = −0.92, SE = 0.12, *p* < 0.001, 95% CI = (−1.11, −0.69)], also classified as a large effect. Additionally, screen time had a significant effect on adolescents' BMI [*β* = 0.08, SE = 0.04, *p* < 0.05, 95% CI = (0.01, 0.15)], with a small effect size. BMI had a significant effect on adolescents' mental health [*β* = −0.50, SE = 0.04, *p* < 0.001, 95% CI = (−0.59, −0.42)], with a large effect size. BMI partially mediates the relationship between screen time and mental health, with an indirect effect value of −0.04 [95% CI = (−0.08, −0.0004)], classified as a small effect. Statistical results are presented in Tables 3, 4, and the structural path is shown in Figure 3 for further details.

**Table 3:** Tests the mediation model.

Variable	BMI					Mental health				
	*β*	se	*t*	LLCI	ULCI	β	se	*t*	LLCI	ULCI
Gender	−0.72	0.12	−6.00**	−0.96	−0.48	−3.12	0.39	−8.00**	−3.89	−2.36
Age	0.23	0.04	6.44**	0.16	0.30	0.39	0.12	3.42**	0.17	0.62
Screen Time	0.08	0.04	2.18*	0.01	0.15	−0.89	0.12	−7.71**	−1.11	−0.66
BMI						−0.50	0.04	−11.69**	−0.59	−0.42
R2	0.01					0.04				
F	27.73**					63.02				
Variable	WC					Mental health				
	*β*	se	*t*	LLCI	ULCI	*β*	se	*t*	LLCI	ULCI
Gender	−6.21	0.59	−10.49**	−7.37	−5.05	−2.97	0.40	−7.47**	−3.75	−2.19
Age	−0.17	0.17	−0.95	−0.51	0.18	0.27	0.12	2.37*	0.05	0.50
Screen Time	−0.17	0.17	−1.00	−0.52	0.17	−0.93	0.12	−8.02**	−1.16	−0.70
WC						−0.03	0.01	−3.74**	−0.05	−0.02
R2	0.02					0.02				
F	37.04**					31.75**				

A: ScreenTime, B: Pittsburgh Sleep Quality Index (PSQI), C: waist circumference (WC), D: body mass index (BMI).

*p < 0.05.

**p < 0.001.

#### 3.3.2 Testing of the WC intermediary model

After controlling for covariates, screen time had a significant direct effect on adolescents' mental health (*β* = −0.92, *p* < 0.001), with a large effect size. When waist circumference was included as a mediating variable, the effect of screen time on adolescents' mental health remained significant (*β* = −0.92, *p* < 0.001), with the effect size still classified as large. However, screen time does not have a significant effect on adolescents' waist circumference (*β* = −0.19, *p* > 0.05), and the path effect of waist circumference on mental health does not form a significant mediating effect, indicating that waist circumference does not have a mediating effect between screen time and mental health. Statistical results are shown in Table 3, and the structural path is shown in Figure 3 for detailed information.

### 3.4 Moderation of the mediating effect

**Figure 4:**
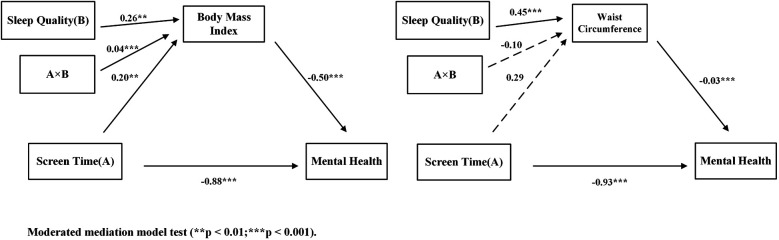
Moderated mediation model test.

**Figure 5:**
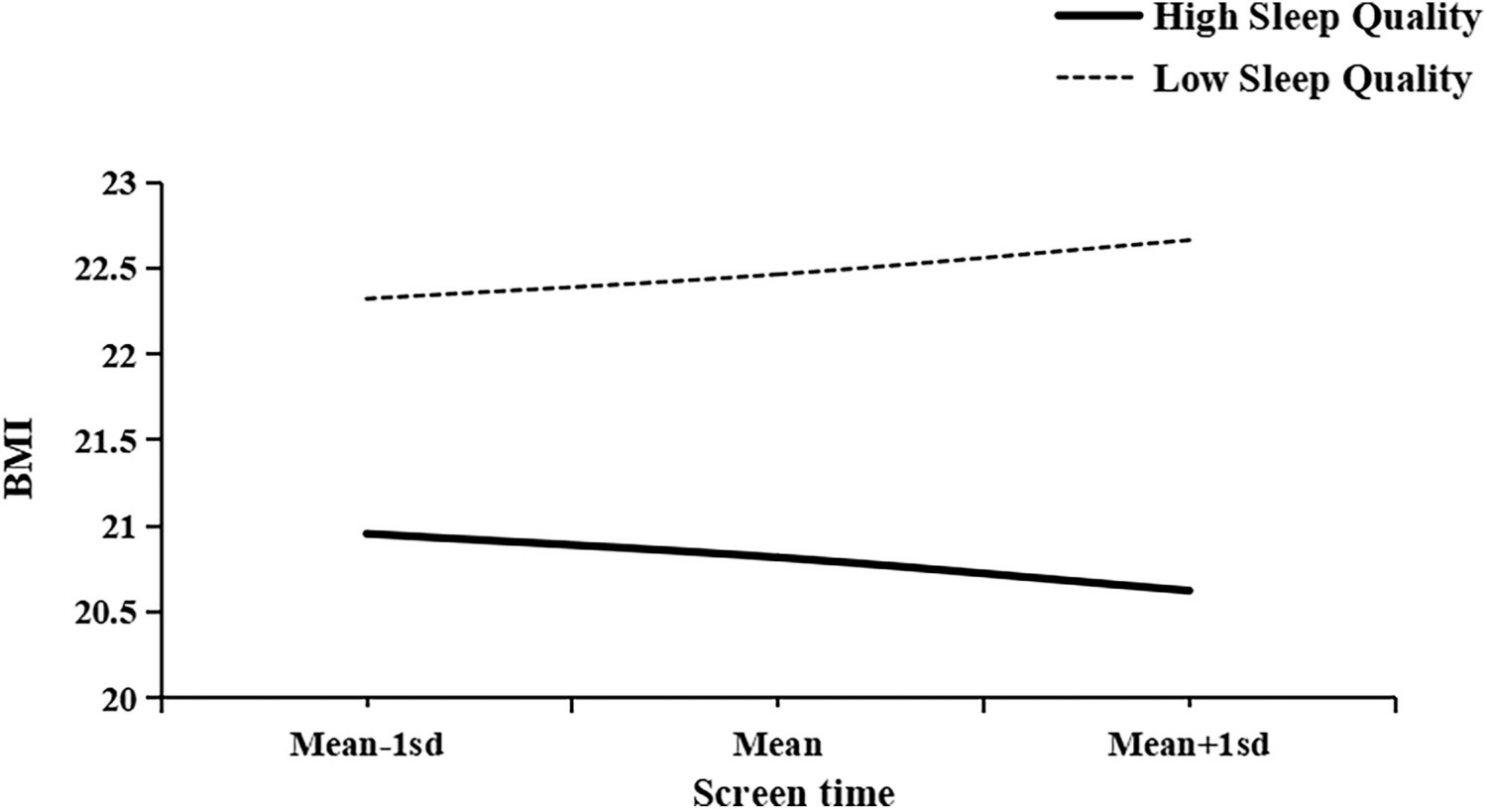
Simple slope plot.

After controlling for covariates, BMI was included as a mediator variable, and sleep quality was included as a moderator variable. Screen time had a significant negative effect on adolescents' mental health [*β* = −0.20, SE = 0.07, *p* < 0.01, 95% CI = (−0.34, −0.06)], with an effect size ranging from small to moderate. Additionally, BMI partially mediated the relationship between screen time and mental health [*β* = −0.89, SE = 0.12, *p* < 0.001, 95% CI = (−1.11, −0.66)], with a large effect size. Sleep quality moderated the relationship between adolescents' screen time and BMI [*β* = 0.04, SE = 0.01, *p* < 0.001, 95% CI = (0.02, 0.07)], with a small effect size. Statistical results are presented in Table 5, structural paths in Figure 4, and moderation slope plots in Figure 5 for detailed information.

**Table 4:** Analysis of mediating effects of BMI.

Effect	Effect size	Bootstrap 95%CI	SE	Proportion of mediating effects (%)
Total effect	−0.93	−1.15, −0.70	0.12	
Direct effect	−0.89	−1.11, −0.66	0.12	95.70
Indirect effect	−0.04	−0.08, −0.0004	0.02	4.30

After controlling for covariates, waist circumference acted as a mediating variable, as shown in Figure 3. Screen time had a significant negative effect on adolescents' mental health [*β* = −0.93, SE = 0.12, *p* < 0.001, 95% CI = (−1.16, −0.70)], with a large effect size; Waist circumference has a significant negative effect on adolescents' mental health [*β* = −0.03, SE = 0.08, *p* < 0.001, 95% CI = (−0.05, −0.02)], with a small effect size. Screen time does not have a significant effect on waist circumference [*β* = 0.29, SE = 0.36, *p* > 0.05, 95% CI = (−0.41, 1.00)], and waist circumference does not have a significant moderating effect on the relationship between adolescents' screen time and mental health [*β* = −0.10, SE = 0.06, *p* > 0.05, 95% CI = (−0.23, 0.02)]. Statistical results are shown in Table 5, and structural paths are shown in Figure 4 for detailed information.

Additionally, to further explore the moderating effect of sleep quality on the relationship between screen time and BMI, this study used simple slope analysis to examine the moderating effects of different sleep qualities (low and high). For adolescents with low sleep quality, screen time had a significant positive effect on BMI [*β*_simple slope = 0.12, SE = 0.05, *p* < 0.05, 95% CI = (0.03, 0.21)], with a small effect size. For adolescents with high sleep quality, screen time had a significant negative effect on BMI [*β*_simple slope = −0.11, SE = 0.05, *p* < 0.05, 95% CI = (−0.2166, −0.0104)], with a small effect size. Statistical results are presented in Table 6, and the slope plots are shown in Figure 5 for further details.

**Table 5:** Moderation and mediation analyses.

Variable	Screen time (h)										Variable	Screen time (h)									
	Body mass index (kg/m^2^)					Mental health						Waist circumference (cm)					Mental health				
	*β*	se	*t*	LLCI	ULCI	*β*	se	*t*	LLCI	ULCI		*β*	se	*t*	LLCI	ULCI	*β*	se	*t*	LLCI	ULCI
Gender	−0.96	0.12	−8.03***	−1.19	−0.72	−3.12	0.39	−8.00***	−3.90	−2.36	Gender	−0.27	0.18	−1.52	−0.62	−0.08	−2.97	0.4	−7.47***	−3.75	−2.19
Age	0.14	0.04	4.02	0.07	0.21	0.39	0.12	3.42	0.17	0.62	Age	−6.46	0.6	−10.8	−7.63	−5.29	0.27	0.12	2.37	0.18	0.5
A	−0.20	0.07	−2.74**	−0.34	−0.06	−0.89	0.12	−7.71***	−1.11	−0.66	A	0.29	0.36	0.81	−0.41	1.00	−0.93	0.12	−8.02***	−1.16	−0.7
B	0.26	0.03	9.30**	0.20	0.31						B	0.45	0.14	3.27**	0.18	0.73					
D						−0.50	0.04	−11.69***	−0.59	−0.42	C						−0.03	0.008	−3.74***	−0.05	−0.02
A × B	0.04	0.01	3.51***	0.02	0.07						A × B	−0.1	0.06	−1.66	−0.23	0.02					
R2	0.05					0.04					R2	0.02					0.04				
F	56.48***					63.02***					F	24.40***					31.75***				

A: ScreenTime, B: Pittsburgh Sleep Quality Index (PSQI), C: waist circumference (WC), D: body mass index (BMI).

**p < 0.01.

***p < 0.001.

**Table 6:** The moderating effect of different sleep quality levels on the relationship between screen time and BMI.

Probing moderated indirect relationships		Standardized coefficient	Lower limit 95%CI	Upper limit 95%CI	*p*
	Sleep quality				
Screen time	High	0.12	0.03	0.20	*p* <0.001
	Low	−0.11	−0.22	−0.01	*p* < 0.05
Index of moderated mediation		−0.022	−0.05	−0.003	*p* < 0.001

## 4 Discussion

This study investigates the relationship between screen time, mental health, BMI, waist circumference, and sleep quality among Chinese adolescents. The study also examines the mediating role of BMI and waist circumference in the relationship between screen time and mental health, as well as the moderating role of sleep quality in the relationship between screen time and BMI and waist circumference. The main findings of this study are as follows: (1) Screen time is positively correlated with BMI, BMI is negatively correlated with mental health, screen time is negatively correlated with mental health, and sleep quality is negatively correlated with BMI. (2) Waist circumference is negatively correlated with mental health, sleep quality is negatively correlated with waist circumference, and there is no correlation between screen time and waist circumference. (3) After controlling for gender and age, BMI mediates the relationship between screen time and mental health in adolescents, while sleep quality moderates the relationship between screen time and BMI. These results validate the initial hypotheses of this study. However, waist circumference does not mediate the relationship between screen time and mental health. Sleep quality does not moderate the relationship between screen time and waist circumference.

### 4.1 Associations between adolescent screen time and mental health

Research has found that screen time is negatively correlated with mental health, a finding consistent with recent longitudinal evidence from other studies. For example, Santos et al. found that social media use is negatively correlated with mental health and pointed out that girls are at higher risk of depression (10). Although our study is cross-sectional and cannot prove causality, evidence from intervention studies supports our findings. For example, a two-week intervention study showed that reducing screen use effectively improved the mental health of children and adolescents (44), providing strong causal evidence for the association between screen time and mental health. However, different studies have varying definitions and measurement methods for screen time and mental health, which may also be a reason for the inconsistent results. For example, some studies define screen time as the total time spent using all electronic devices (45), while others focus only on specific activities, such as social media or video games (46). This study used self-report questionnaires, which may be subject to recall bias. In addition, a meta-analysis found a weak but significant correlation between screen time and internalizing and externalizing behavior problems in children (45). In summary, despite methodological differences, the findings of this study are consistent with the mainstream literature, namely that excessive screen time has a potentially negative impact on adolescents' mental health.

The negative correlation between screen time and mental health found in this study can be further understood from the perspective of Bronfenbrenner's ecological systems theory (47). This theory emphasizes that individual development is influenced by multiple environmental systems at different levels. At the microsystem level, prolonged screen use may reduce face-to-face interaction between adolescents and their families and peers, thereby weakening important social support (48). At the mesosystem level, excessive engagement in online gaming or social media may impair sleep quality, which in turn affects academic performance and may lead to more profound mental health issues (49). At the broader external and macro system levels, parental work stress (50) and family economic status (51) and the widespread acceptance of technology and social media in mainstream society (52) may indirectly influence adolescents' screen usage patterns. The impact of screen time on adolescent mental health is not a single-path phenomenon, but rather the result of complex interactions and interplay across multiple levels, including individual, family, peer, school, and sociocultural factors. Our research findings align with this theoretical framework, indicating that to fully understand and effectively address this issue, it is necessary to adopt multi-level, systematic intervention measures rather than focusing solely on individual behavior.

### 4.2 The mediating role of BMI

This study found a positive correlation between screen time and BMI. The use of electronic devices can help adolescents alleviate stress and anxiety, but prolonged screen time may lead to internet addiction (53) and is associated with sedentary behavior, where the body is in a low-energy state and lacks physical activity (54) and increase the risk of overweight and obesity (55). This study also found a positive correlation between BMI and mental health. Research indicates that a higher BMI may lead to negative body image perceptions among adolescents, which in turn may affect their self-esteem and social interactions, thereby exacerbating mental health issues (56). Body image is closely related to self-esteem, especially in early adolescence, when this relationship is particularly evident (57). At this stage, adolescents are undergoing rapid physical changes and seeking social acceptance. Low self-esteem and psychological distress are often significantly related to dissatisfaction with body shape (58). A negative body image not only affects adolescents' self-confidence, but can also lead to social media addiction, further exacerbating mental health issues such as anxiety and depression (58).

This study found that BMI mediates the relationship between screen time and mental health in adolescents, consistent with similar findings in previous studies (59). Based on social cognitive theory and self-determination theory, we can gain a deeper understanding of the psychological mechanisms underlying this phenomenon. Social cognitive theory emphasizes the interdependent relationship between individuals, behavior, and the environment (60). Increased screen time is associated with sedentary behavior, often accompanied by reduced physical activity and insufficient energy expenditure, leading to an increase in BMI (61). A high BMI further affects adolescents' physical self-efficacy and body image perception (62), making them more prone to negative emotions and psychological distress (63). Therefore, BMI is not only a physiological indicator, but also indirectly affects mental health through cognitive and emotional mechanisms. Secondly, self-determination theory suggests that an individual's mental health depends on the fulfillment of basic psychological needs (autonomy, competence, and relatedness) (64). Excessive screen use often limits physical activity and real-life social opportunities, thereby weakening adolescents' sense of competence and social connection experienced through physical activity (65). An increase in BMI may further reduce motivation to participate in physical activity and social occasions, making it more difficult to satisfy basic psychological needs and ultimately having a negative impact on mental health (66).

In summary, screen time has a direct negative impact on adolescents' mental health and indirectly affects their mental health through BMI. BMI plays a partial mediating role in this relationship, but the indirect effect is small, indicating that its impact is relatively limited. Nevertheless, even small effects can have significant implications for public health in large sample populations. Previous studies have shown that excessive screen time among adolescents is associated with an increased risk of obesity (19), leading to higher BMI among adolescents. Adolescents with higher BMIs are more likely to experience social withdrawal (67), social isolation (68), bullying (69), and humiliation (70). Adolescents with higher BMIs may develop mental health issues. Therefore, the results of this study confirm Hypothesis 2, namely that BMI plays a partial mediating role in the relationship between screen time and mental health in adolescents.

However, this study found that waist circumference did not mediate the relationship between screen time and mental health. There was no significant correlation between adolescents' screen time and waist circumference, which is inconsistent with the findings of Padmapriya et al. (22), who found that longer screen time in adolescents was associated with higher levels of abdominal obesity. In addition, a meta-analysis showed that there was no significant association between screen time and abdominal obesity in adolescents. Although the waist circumference of the high screen time group was significantly higher than that of the low screen time group, the difference was small (71). The impact of screen time on waist circumference is not fixed and may be influenced by various factors, such as diet and sleep (72). In contrast, BMI was significantly associated with screen time and mental health in this study. This may be because BMI reflects the overall balance between energy intake and expenditure, which is more directly influenced by diet and physical activity. Waist circumference, as an indicator that concentrates on abdominal fat distribution, is often more strongly influenced by genetic, hormonal, and metabolic factors (73, 74). During the early developmental stage of adolescence, energy allocation mechanisms tend to favor overall weight gain rather than localized fat deposition. During this stage, growth hormone is active and subcutaneous fat storage capacity is strong, while the biological mechanism for visceral fat accumulation has not yet been fully activated (75, 76). This may explain why screen time is significantly positively associated with BMI, but not statistically significantly associated with waist circumference. Therefore, in short-term or cross-sectional studies, the association with screen time may not be as obvious as that with BMI. Future studies should combine longitudinal designs and consider dietary, sleep, and metabolic factors to further explore the differential roles of BMI and waist circumference in the mechanisms by which screen time affects mental health.

### 4.3 Moderated mediating effect of sleep quality

This study found that sleep quality moderates the relationship between screen time and BMI, which is consistent with Hypothesis 3. Sleep quality not only positively predicts adolescents' BMI, but also moderates the effect of screen time on BMI. Specifically, as shown in Figure 4, poor sleep quality in adolescents exacerbates the negative impact of screen time on BMI, and excessive screen time in adolescents with poor sleep quality may increase BMI. This result is consistent with Danil's findings: screen time has a more significant impact on BMI among adolescents who sleep less (77). Although this study did not directly examine sleep duration, sleep quality and sleep duration are closely related. Research shows that shorter sleep duration negatively affects an individual's sleep quality, leading to difficulty falling asleep or frequent waking during the night (78). Conversely, poor sleep quality can also make it difficult for individuals to get adequate sleep duration (79). According to circadian rhythm theory (80), human metabolism and appetite control processes are regulated by biological clock rhythms. Adolescents with poor sleep quality may experience circadian rhythm disorders due to reduced melatonin secretion, which can lead to metabolic abnormalities, increased appetite, and hunger, ultimately resulting in weight gain (81). If teenagers stare at electronic screens for long periods of time, especially at night, the blue light emitted by the screens may suppress melatonin secretion (82). Reduced melatonin secretion is associated with insulin resistance, high uric acid levels, and elevated triglyceride levels, which may lead to obesity in adolescents (83). In addition, reduced melatonin secretion may interfere with the breakdown and synthesis of adipose tissue, thereby exacerbating obesity (84). Another explanation is that poor sleep quality may be a source of stress (56), which could exacerbate overeating caused by stress (85). Lack of sleep can cause fatigue in adolescents (86), which in turn reduces physical activity levels and leads to overweight and obesity. In addition, poor sleep quality may trigger increased appetite (87) and energy metabolism disorders (88), further leading to an increase in BMI. However, if an individual has high-quality sleep and a stable circadian rhythm, their physiological functions are more likely to remain balanced (89), thereby mitigating the negative impact of screen time on BMI. Sleep quality mediates the relationship between screen time and BMI. Although the effect size is small, the results suggest that good sleep quality can partially buffer the adverse effects of screen time on BMI. For adolescents with poor sleep quality or long screen time, interventions to improve sleep should be prioritized to reduce their risk of obesity.

### 4.4 Limitations and implications

This study has both theoretical and practical significance. The theoretical significance of this study lies in providing a theoretical framework for the interaction of multiple factors. The research findings contribute to understanding the causes of mental health, reveal the complexity of mental health issues, and offer a new perspective on understanding adolescent mental health. Additionally, the findings of this study provide guidance for prevention and intervention strategies and serve as a reference for policy-making. The practical significance of preventing and intervening in mental health issues is as follows: first, adolescents should use electronic devices reasonably to reduce dependence and excessive use. Second, adolescents should increase their physical activity levels; if adolescents spend excessive time on screens, we recommend that they engage in at least 70 min of moderate-to-vigorous physical activity daily to reduce body fat (90). Finally, adolescents should establish regular sleep patterns to ensure adequate sleep duration and quality, thereby lowering the risk of mental health issues.

However, this study has certain limitations. First, this is a cross-sectional study and cannot explain the causal relationships between variables. To address this limitation, future studies should adopt longitudinal or experimental designs to better understand the causal pathways and dynamic interactions of these variables over time. Such studies will enhance the robustness and applicability of research findings and provide stronger evidence for developing targeted intervention measures. Second, sleep quality, screen time, and mental health were measured through questionnaire surveys, and the reliability of self-reported data may influence the results. Future studies should incorporate more objective measurement methods (e.g., wearable devices, digital tracking data) to enhance the validity of the results. Third, the study did not distinguish between different types of screens, making it impossible to determine which type of screen use (e.g., social media, video games, educational software, etc.) affects adolescents' mental health and obesity. Future studies should further refine the types of screen use and explore the mechanisms by which different types of screens affect adolescents' mental health and obesity, thereby providing a basis for developing more precise intervention measures. Fourth, this study did not fully account for the nested structure of the data. Future research should employ methods such as multilevel models to more comprehensively analyze the relationships among these variables.

## 5 Conclusions and recommendations

This study investigated the relationship between screen time, mental health, BMI, and sleep quality among Chinese adolescents. The results revealed that BMI mediated the relationship between screen time and mental health, while sleep quality moderated the relationship between screen time and mental health. Furthermore, the study found that sleep quality exerted varying degrees of moderating effects on the relationship between screen time and adolescents' BMI. Improving sleep quality weakened the association between screen time and adolescents' mental health. These findings suggest that simply limiting adolescents' screen time may not be sufficient to effectively improve mental health; improving sleep quality is also a key strategy. Future interventions should focus on a multidimensional, coordinated approach that addresses physical, psychological, and behavioral factors. Education and public health departments can incorporate sleep education, healthy screen use, and weight management into school health programs. Schools can reinforce healthy screen use and good sleep habits through curricula and behavioral guidelines, while families should monitor screen time and encourage regular sleep schedules. Collaboration between schools, families, and communities can effectively promote adolescent mental health. Future research should adopt a longitudinal, multi-time point tracking design to explore the causal relationship and long-term dynamic effects between screen time, BMI, sleep quality, and mental health, and combine intervention studies to test whether improving sleep quality and healthy behaviors can buffer the adverse effects of excessive screen time.

## Data Availability

The raw data supporting the conclusions of this article will be made available by the authors, without undue reservation.
